# Sexual size dimorphism, prey morphology and catch success in relation to flight mechanics in the peregrine falcon: a simulation study

**DOI:** 10.1111/jav.01979

**Published:** 2019-03-20

**Authors:** Robin Mills, Graham K. Taylor, Charlotte K. Hemelrijk

**Affiliations:** Groningen Inst. for Evolutionary Life Sciences, Univ. of Groningen, Groningen, the Netherlands; Groningen Inst. for Evolutionary Life Sciences, Univ. of Groningen, Groningen, the Netherlands

**Keywords:** aerial attack, aerodynamics, peregrine falcon, sexual dimorphism

## Abstract

In common with many other raptors, female peregrine falcons *Falco peregrinus* are about 50% heavier than males. Their sexual dimorphism is thought to allow breeding pairs to exploit a wider range of prey through a division of labor: the male being able to catch more maneuverable prey species; the female capable of carrying larger ones. Given the difficulty of assessing the catch success and load carrying capacity of both sexes of falcon in the field, we here adopt a novel approach to test the division-of-labor theory by using a detailed physics-based flight simulator of birds. We study attacks by male and female peregrines on prey species ranging from small passerines to large ducks, testing how catch success relates to the flight performance of predator and prey. Males prove to be better than females at catching highly maneuverable prey in level flight, but the catch success of both sexes improves and becomes more similar when diving, because of the higher aerodynamic forces that are available to both sexes for maneuvering in high-speed flight. The higher maximum roll acceleration of the male peregrine explains its edge over the female in catching maneuverable prey in level flight. Overall, catch success is more strongly influenced by the differences in maneuverability that exist between different species of prey than between the different sexes of falcon. On the other hand, the female can carry up to 50% greater loads than the male. More generally, our detailed simulation approach highlights the importance of several previously overlooked features of attack and escape. In particular, we find that it is not the prey’s instantaneous maximum centripetal acceleration but the prey’s ability to sustain a high centripetal acceleration for an extended period of time that is the primary driver of the variation in catch success across species.

## Introduction

Peregrine falcons *Falco peregrinus* – hereafter referred to as peregrines – are the world’s most widely distributed raptor (Ferguson-Lees and Christie 2001). They hunt a wide variety of avian prey using a range of alternative attack strategies (Rudebeck 1951, Dekker 1988, Dekker and Taylor 2005, Zoratto et al. 2010). In nature, wild peregrines take prey ranging from small passerines such as goldcrests *Regulus regulus* and Eurasian blue tits *Cyanistes caeruleus* (Ratcliffe 2010), up to larger waterfowl such as mallards *Anas platyrhynchos* (Dekker 2009), spanning well over two orders of magnitude in body mass. The only species that are not attacked are very large birds such as swans and geese (but see Ratcliffe 2010), although the medieval kings of England successfully trained falcons to hunt quarry as large as grey herons *Ardea cinerea* and common cranes *Grus grus* (Oggins 2004). Likewise, forest-dwelling species are rarely taken because the peregrine requires wide open spaces for its preferred hunting modes (Ratcliffe 2010), including the famous stoop in which the falcon soars to a high altitude before diving down at great speed to intercept its prey in mid-air. The peregrine’s choice of prey appears to be opportunistic and dependent on the availability of different prey species (Stevens et al. 2009), which varies by habitat and time of year, but many reports suggest a bias towards certain species, even after accounting for their availability (Ratcliffe 2010). The peregrine is particularly well known for taking pigeons, notably rock doves *Columba livia* and their feral counterparts – or when these are scarce, for taking other species of a similar size, such as black-headed gulls *Larus ridibundus* (Kruuk 1964). The majority of its preferred prey species weigh between 0.05 and 0.5 kg (Ratcliffe 2010). Interestingly, there is a marked difference in the choice of prey between male and female peregrines (Dekker 2009). Males consistently bring smaller prey back to the nest (Parker 1979) and are more often observed to hunt for small passerines (e.g. common starlings *Sturnus vulgaris)* and small waders (e.g. dunlins *Calidris alpina)*, whereas females hunt more often for larger birds up to the size of ducks (e.g. northern pintails *Anas acuta)* (Dekker 1980, 1987, 2009).

It has been theorized that the reversed sexual size dimorphism of falcons has evolved such that a pair of birds has a broader selection of prey to choose from (Dekker 2009). The heavier female is supposed to be able to carry larger prey, and the smaller, lighter male is supposed to be more adept at attacking highly maneuverable prey, but it is hard to judge from empirical data whether the relative ease of catching different prey species underlies the observed differences in prey choice between the sexes – not least because the sex of a falcon is hard to identify when observing a high-speed chase (Dekker 2009). In addition, the empirical relationship between catch success and flight ability is hard to investigate, because the reported success rates of peregrines vary greatly between studies. In one study (Jenkins 2000), catch success was reported to be highest for small doves, and small passerines such as sparrows and queleas; intermediate for small to medium sized birds such as starlings and weavers; and lowest for large pigeons, ducks and waders. In another study (Dekker 2009), the opposite pattern was observed: high success rates for ducks and waders; intermediate for gulls; and lowest for small passerines. Environmental variation aside, one underlying cause of these conflicting findings may be the differing intensity with which the falcons hunted. Many of a falcon’s attacks do not appear genuinely intended to kill them (Ratcliffe 2010); perhaps because the falcon is warming up, is playing, is practising or had eaten enough before the attack that its motivation is low. To account for this varying motivation, the terms high-versus low-intensity attacks have been introduced in the literature (Treleaven 1980), where the intensity is judged visually by the observer, but such classification remains subjective. Most problematically, it has been observed that male falcons are more motivated to catch smaller prey than females (Dekker 2009), thereby obscuring any differences in their actual ability to catch them.

**Figure 1:**
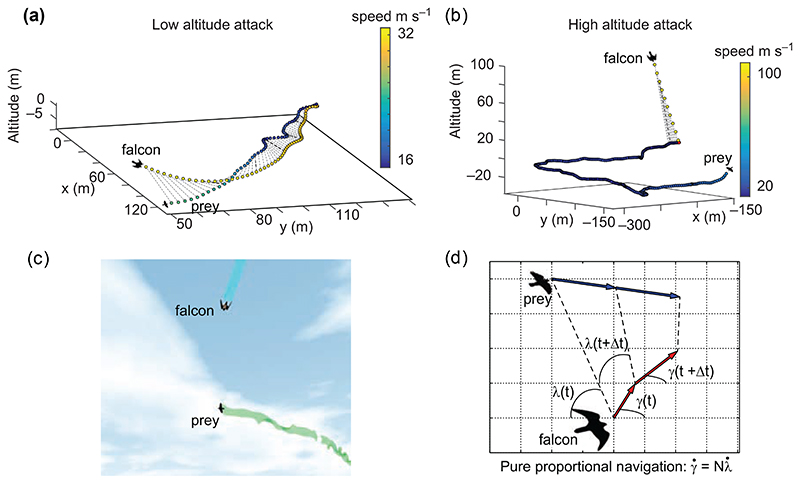
Visualisation of the bird flight simulator. (a) Low-speed attack by a falcon. The dotted grey lines connect the positions of the falcon and prey at a given time point with an interval of 100 m s. If the falcon initiates its attack from only 50 m above the prey, then it reaches a speed of approximately 30–40 m s^–1^ near intercept. Prey in the model fly erratically, exerting high accelerations to the left and right. (b) High-speed stoop by a falcon on its prey. By initiating its attack from 1500 m above the prey, and by retracting its wings, the falcon is able to reach speeds of over 100 m s^–1^. (c) Graphical output of the simulator. The real-time graphical output of the simulator shows how falcon and prey adjust their wingbeat, wing retraction, body orientation and trajectory. The colored lines behind the birds denote their trajectories. (d) A graphical depiction of the pure proportional navigation guidance law of the peregrine. Under this guidance law, the commanded angular rate of change in the falcon’s velocity is directly proportional to the angular rate of the line-of-sight between the falcon and its prey.

The difficulties inherent in studying the factors affecting catch success through field observation motivate us to apply a new and different approach here, using a physics-based bird flight simulator to study the problem in silico (Fig. 1, Supplementary material Appendix 1). The mathematical details of the flight simulator are summarised here in Supplementary material Appendix 1 and are explained fully in Mills et al. (2018). To allow meaningful inference from the simulation results, the model aims to capture all the key dynamics of the predator–prey scenario. Specifically, it simulates the mechanics and aerodynamics of flapping and gliding flight by both birds, the control mechanisms by which the model birds manipulate their aerodynamic forces, the guidance law by which the model falcons determine how to intercept their prey, and the visual system providing feedback to this guidance law. It has been shown experimentally that the flight trajectories of peregrines follow a guidance law called pure proportional navigation (Brighton et al. 2017). Under this guidance law, the commanded angular rate of change in the falcon’s velocity is directly proportional to the angular rate of the line-of-sight between the falcon and its prey (Fig. 1d). Our model falcons use the same guidance law, exhibiting a realistically short response delay to maneuvers of the model prey, whose motion they observe with a small degree of visual error. Appropriate tuning of this guidance law is crucial for accurate interception, which we model by optimizing the constant of proportionality in the guidance law (see Mills et al. 2018 for a theoretical exposition and Brighton et al. 2017 for an empirical investigation on proportional navigation in peregrines). Varying this so-called navigation constant (*N*) manipulates the trade-off between higher steering effort and faster convergence to a collision course, as well as influencing the precision of steering in the presence of error. Each simulated species has a different set of morphological parameters (i.e. body mass, moment of inertia, wing area, wingspan, wingbeat frequency, etc.), which in turn determines the mechanical and aerodynamic limits on force production, and hence the ability of the bird to accelerate and maneuver. We simulate attacks of lone falcons intercepting lone prey in mid-air, and parametrically vary the starting altitude of the falcon and its starting distance to the prey, so as to mimic the variety of attack strategies that real peregrines use – from level chases to stooping. By running these variations in a Monte Carlo simulation, we test whether the optimal attack strategy differs between male and female falcons, and whether the optimal attack strategy depends upon prey species.

Real prey use a variety of escape strategies, the most ubiquitous of which our simulations attempt to capture. Most ducks drop ‘like a falling stone’ towards the nearest body of water before submerging (Dekker 1980), sometimes reaching a dive speed matching that of the falcon (Dekker 2009). If the duck cannot reach safety before the falcon has caught up, it will maneuver erratically at the last moment (Dekker 2009). Other prey instead aim high in the sky (Lima 1993): many passerines can out-climb a falcon, and are therefore safe once they reach a slightly higher altitude than the predator. When alerted that a predator is present, many species will start to fly fast and erratically, maneuvering in a way that appears to make it hard for the falcon to catch them. Such behavior is mainly observed in isolated individuals under attack (Kruuk 1964), but is also seen in groups. For instance, the fast, alerted flight of a flock of common starlings manifests itself as dark waves in the murmuration, which are thought to be caused by distinctly-timed and synchronized zig-zags on the part of the individuals within the flock (Hemelrijk et al. 2015). Erratic, or ‘jinking’, flight seems to represent an invaluable adaptation for predator avoidance, because non-alerted, straight-flying prey are almost always caught (Dekker 1980). This erratic flight mode, with distinctly timed strong acceleration to either side, is fascinating from a theoretical standpoint as it turns out to be the optimal escape strategy when evading missiles that use the same pure proportional navigation guidance law as attacking peregrines (Girard and Kabamba 2015). Yet this jinking escape pattern has never been studied in birds, and previous research has instead focused on studying escape by climbing, diving or turning smoothly (Hedenström and Rosén 2001). We therefore also study the importance of erratic maneuvering by the prey in our physics-based simulation of aerial attack behaviors.

In summary, our aim in this paper is to provide a comprehensive analysis of how catch success in peregrines is affected by their own flight performance and by that of their prey, and to use this to explore whether intersexual variation in flight performance is expected to lead to intersexual variation in catch success against different prey species. We do this in a simulation environment that accurately captures the physics of the situation, and in which the behavior of predator and prey is optimized to respectively maximize or minimize catch success.

## Methods

This paper reports the results of three in silico experiments. In our first experiment, we investigate the catch success of model falcons against a variety of model prey species differing in morphology and flight performance. Our aim in this experiment is to improve our understanding of prey choice in peregrines, and to test whether male falcons are indeed more adept than females at catching highly maneuverable prey. In our second experiment, we study which specific properties of the prey species make them hard to catch, while in a third experiment, we investigate the properties of the falcon that enable successful capture.

### Simulation model

The simulation represents individual birds in an open, three-dimensional space, thereby simulating a scenario in which predator and prey fly at high-altitude. Model birds have six degrees of freedom in movement: three in translation, and three in rotation. These rotations and translations are produced by gravitational and aerodynamic forces in a physics-based model of the flight dynamics. Model prey maneuver erratically under a random guidance command with directional bias (Supplementary material Appendix 1), while model falcons aim to intercept their prey in a single attempt using the guidance law pure proportional navigation. This guidance law feeds back the angular rate of the line-of-sight between the falcon and its prey, which is assumed to be measured with error by the visual system (Supplementary material Appendix 1). The guidance system controls turning by commanding an acceleration normal to the bird’s velocity vector. How closely the bird is able to meet this guidance command is determined in one of two ways, assuming that the bird uses bang–bang control to redirect its aerodynamic force vector as quickly as possible when banking to turn. The aerodynamic forces and moments are assumed to be generated by flapping or gliding, using variable wing retraction as a control (Supplementary material Appendix 1). A quasi-steady blade-element model is used to solve for the maximum lift force that can be produced normal to the bird’s velocity vector at a given airspeed, and for the maximum thrust (or minimum drag) force that can be produced perpendicular to the lift by flapping or gliding at any given sub-maximal value of lift production. The aerodynamic forces that the birds can exert are limited by the stall threshold and by mechanical constraints. When a bird cannot exert the acceleration commanded by its guidance law, it exerts the maximum lift it can to turn, and minimizes the corresponding drag.

This approach captures how the bird’s flight morphology impacts its flight performance, and hence its success at achieving or evading capture, on the assumption that the bird’s immediate objective is to fly as fast as it can, subject to meeting the acceleration demands of its guidance system as closely as possible (see below). We justify this assumption in two ways. First, Howland (Howland 1974) shows that a fleeing prey organism can escape a predator by turning more tightly than its pursuer, if and only if $v>\sqrt{r}$, where *v* is the speed of the prey normalized by the speed of the predator, and *r* is the turning radius of the prey normalized by the turning radius of the predator. Making use of the fact that centripetal acceleration is equal to speed squared divided by turn radius, it follows that we can rewrite Howland’s inequality as *a* > 1, where *a* is the centripetal acceleration of the prey normalized by that of the predator. The available centripetal acceleration of any bird increases with its airspeed on account of the greater aerodynamic forces produced, which implies that both predator and prey should aim to maximize their flight speed when turning, so as to maximize and minimize the falcon’s catch success respectively. Second, simulations of missile behavior show that their miss distance decreases as their maximum centripetal acceleration increases, and increases as target acceleration increases (Shneydor 1998, Palumbo et al. 2010). This implies that both attacker and attacked should aim to maximize forward speed when turning. Our model birds therefore flap when the forward component of their acceleration is maximized by flapping, and else glide with the amount of wing retraction that minimizes their drag (Taylor et al. 2016).

To identify which particular aspects of flight performance are important to achieving or evading capture in our model, we also adopt a second approach in which the physics of aerodynamic force production are ignored by dropping the blade-element modelling. In this case, the bird is instead assumed to fly at a given constant flight speed, with a fixed upper limit on the maximum lift force and roll moment it can produce. By varying these limits parametrically, we explore the effects of each aspect of flight performance separately.

### Genetic algorithms

**Figure 2:**
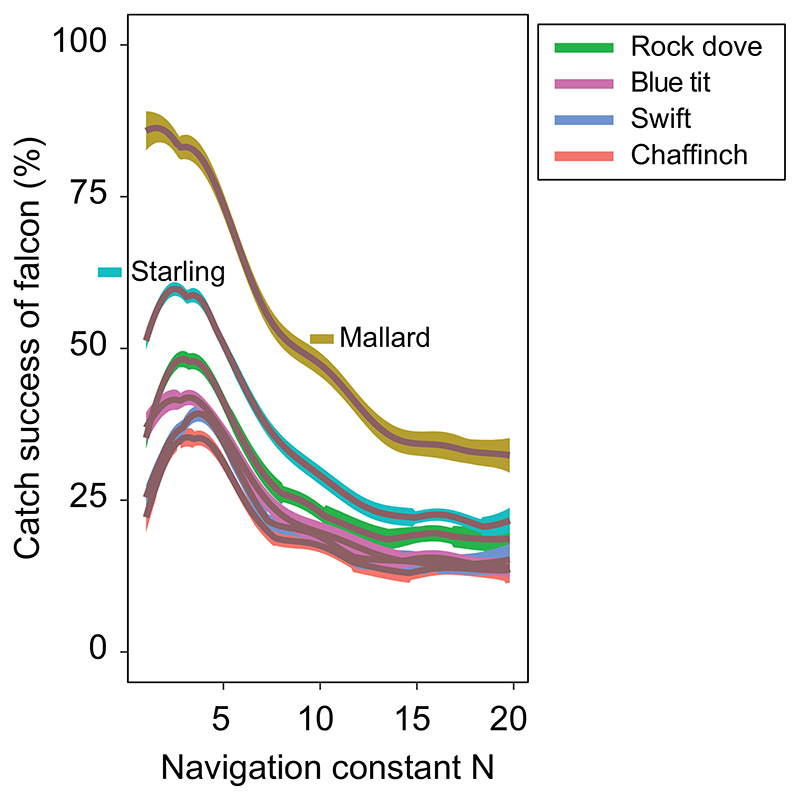
Example of a fitness landscape using genetic algorithms. The catch success (i.e. fitness) of a male peregrine is shown here as a function of the navigation constant *N*, which is the sole free parameter of its pure proportional navigation guidance law. The catch success depicted here is conditional upon the falcon beginning its attack from the starting position representing the best of its three alternative attack strategies (i.e. high altitude, moderate altitude, low altitude). The setting of *N* that maximizes catch success is *N* ≈ 3 for all prey species, with the exception of the mallard, for which lower values of *N* increase catch success.

Several of the model’s guidance parameters are optimized using genetic algorithms (Grefenstette 1986, Holland 1992), prior to running the final sets of simulations whose results we report. First, we optimize the free parameters (*c_1_* ..., *c*_4_) of the guidance function generating erratic prey maneuvers (Supplementary material Appendix 1). Here, the centripetal acceleration of the prey serves as the fitness function, which is independent of the predator’s flight behavior. Second, we optimize the navigation constant (*N*) of the falcon’s pure proportional navigation guidance law (Fig. 1d, 2). In this case, catch success serves as the fitness function, which is obviously conditional upon the optimized flight behavior of the prey. Finally, we re-run the simulations to compute catch success for these optimized values of the guidance parameters. In each generation, the fittest half of the population reproduces two copies of itself, with and without mutation. We apply haploid inheritance with Gaussian mutation on the log scale of the evolving parameter:

(1) $$\text{log}\;g_{i}(n+1)=\text{log}\;g_{i}(n)+\mu$$

where *g* is the mutating parameter, *n* the generation and μ ~ *𝒩* (0, σ^2^). By varying σ and the initial values of the parameters, we find consistently the same optima.

### Experimental design

In each simulation, the prey starts at the origin of the simulation space, with its body in a random orientation, while the falcon starts at a certain horizontal and vertical distance from the origin, heading initially towards its prey. The initial speeds of the falcon and its prey are those which minimize their respective cost of transport (i.e. their maximum-range speeds), and the prey flies for 10 s before the falcon initiates its attack. This simulates a scenario in which the prey has spotted the falcon, and has established erratic flight behavior in response. The simulation runs until the falcon intercepts its prey, or until a near-miss occurs. An intercept is defined as occurring when the predator’s distance to its prey is < 0.2 m. A near-miss occurs when the predator comes within 5 m of its prey, but then finds its prey within its blind zone, defined as a 90° spherical wedge pointing opposite to its heading.

#### Experiment 1

In experiment 1, we aim to identify the optimal attack strategy for each sex of peregrine against a variety of prey species: model falcons therefore have the morphological attributes of either a male or female falcon, whereas model prey have the morphological attributes of one of the six male prey species in Table 1, spanning two orders of magnitude in body mass. In this experiment, model falcons begin their attack from one of three altitudes, representing three alternative attack strategies: high altitude (1500 m above the prey, at a horizontal distance of 50 m), moderate altitude (200 m above the prey, at a horizontal distance of 100 m), and low altitude (50 m above the prey, at a horizontal distance of 200 m). The three initial positions are selected because they span the empirical variation in attacks by falcons (and see Mills et al. 2018 for attack success in intermediate initial positions of a male falcon attacking a starling).

#### Experiment 2

**Figure 3:**
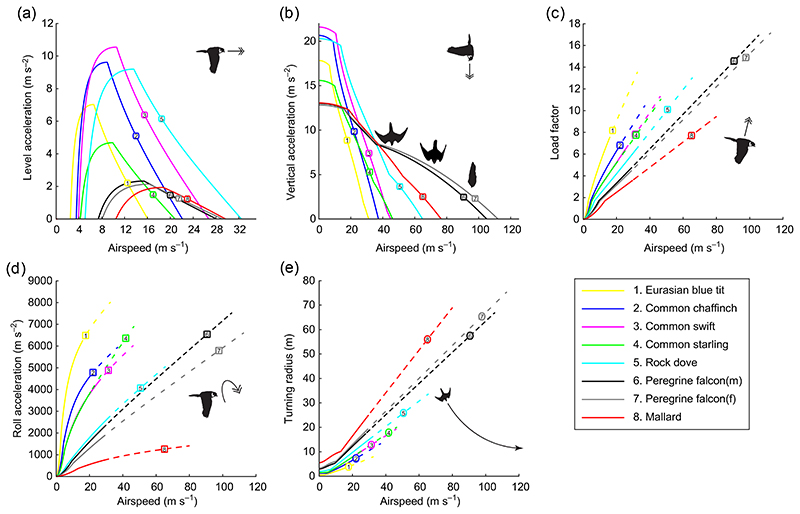
Flight performance of both sexes of peregrine, and species of prey. (a) Maximum available level acceleration versus airspeed, assuming that lift balances weight. Top speed is reached at the maximum airspeed for which the level acceleration is equal to or greater than zero. (b) Maximum vertical acceleration versus airspeed in a vertical dive. (c) Maximum load factor (lift divided by weight) versus airspeed. The solid lines denote the speeds attainable by the bird in level flight; dashed lines denote speeds that are only attainable when diving, and end at the bird’s maximum dive speed. (d) Maximum roll acceleration versus airspeed. (e) Minimum turning radius versus airspeed.

In experiment 2, we study how the prey’s flight performance impacts the catch success of the falcon. The key issue here is that we cannot directly study which specific aspects of flight performance underlie variation in catch success in experiment 1, because the various flight performance attributes are all physically related. For instance, the centripetal acceleration that a bird can apply to achieve a steady banked turn depends on the total amount of lift that it can exert relative to body weight. It follows that the load factor, defined as lift divided by weight, is a key flight performance metric in steady maneuvering. Likewise, the angular acceleration that a bird can apply to roll into a turn depends on the lift asymmetry that it can sustain between its wings, this time expressed relative to its roll moment of inertia. Clearly, the extent of the available lift asymmetry will be closely related to the bird’s maximum load factor, so a prey species that can sustain the high load factors needed to achieve high steady turning performance will also be able to attain the high roll accelerations needed to achieve high unsteady flight performance. Furthermore, a bird’s ability to maneuver is closely related to its flight speed (Fig. 3), with a different relationship for each species. Our approach is therefore to remove any correlation between the different dimensions of flight performance artificially, by allowing the prey’s load factor and roll acceleration to vary freely up to some arbitrary upper limit at some given, but possibly very high, speed. Specifically, we run simulations in which a male falcon attacks prey that fly erratically with a constant forward speed, uniformly sampling 10^6^ times in a three-dimensional parameter space, in which the prey’s flight speed is from 0 to 100 m s^−1^, its maximum load factor from 0 to 15, and its maximum roll acceleration from 0 to 8000 m s^−2^). In this way, we relate the catch success of the falcon to the prey’s flight speed, load factor and roll acceleration independently, using general additive modeling (GAM) to interpolate between the randomly sampled values (Hastie and Tibshirani 1990, Lin and Zhang 1999).

#### Experiment 3

In experiment 3, we investigate how the predator’s flight performance impacts its catch success, by simulating attacks on a blue tit or mallard by a generic raptor with a fixed flight speed, fixed maximum load factor, and fixed maximum roll acceleration. We chose these prey species as being the smallest and largest in our dataset, and therefore differing the most in terms of their flight performance. Again, we uniformly sample values of speed, maximum load factor and maximum roll acceleration 10^6^ times in a three-dimensional parameter space, and apply GAMs to interpolate between the randomly sampled values.

### Statistical analysis

In experiment 1, we perform 10^5^ simulations for each combination of sex of falcon, attack strategy and prey species, such that the widest 95% confidence interval bounds of the catch success are narrower than 0.1%, which guarantees that all visually observable differences in the plots are statistically significant. In the general additive models (GAMs) of experiment 2 and 3, the load factor (*L*), roll acceleration (*R*) and speed (*S*), are the independent variables and catch success (*C*) is the dependent variable:

(2) $$\text{log}(E(C_{i}))=s(L_{i})+s(R_{i})+s(S_{i})+s(L_{i},R_{i},S_{i})+\epsilon$$

where log() is the logit-link function and *s*() is a smooth function estimated by penalized likelihood maximization (Wood 2011). We use generalized cross-validation to optimize the smoothing for predictive accuracy. We did not apply constraints on the effective degrees of freedom.

### Software

The open source bird flight simulator is written in C++ and is available via <https://gitlab.com/BirdFlightSimulator/BirdFlightSimulation>. Matlab 2016b was used for the flight performance analysis, and R statistics (R Core Team) was used for general additive modeling by applying the mgcv package (Wood 2011).

### Data deposition

Data available from the Dryad Digital Repository: < https://doi.org/10.5061/dryad.9m42814> (Mills et al. 2019).

## Results

The outcome of the predator–prey interactions that we model depends closely upon the flight performance of the birds, and we therefore begin by exploring the key parameters of flight performance of the species we model as the backdrop to the rest of the Results, where we consider the outcomes of the three simulation experiments in detail.

### Comparative flight performance of falcons and their prey

Considering the falcon, there is a marked reversed sexual dimorphism (Table 1). Male peregrines are on average two-thirds the body mass (*m_b_*) of females. In combination with the fact that body width typically scales $w_{b}\propto m_{b}^{0.35}$ (Nudds and Rayner 2006), the greater size of the female means that she has a higher overall roll moment of inertia to overcome when maneuvering. However, she also has a greater wing span and area, such that the flight musculature has a larger attachment area on the wings, thereby increasing the maximum aerodynamic torque that she can hold (Tucker 1998, Shyy et al. 2013). This increased maximum torque allows the female to produce larger lift, thrust and roll torque. Furthermore, because the parasite drag on the body scales with body frontal area, which in turn scales as $S_{b}\propto m_{b}^{0.68}$ (Nudds and Rayner 2006), the deceleration due to parasite drag is lower for the female at a given airspeed, such that she reaches a higher terminal speed than the male while diving under gravitational acceleration with fully retracted wings. On the other hand, as the aspect ratio of the female’s wings is lower than that of the male, she encounters a higher induced drag in both flapping and gliding flight. Our aerodynamic model quantifies the net effect of these differences in morphology on the flight performance of both sexes of falcon (Fig. 3; see Table 1 for morphological parameters). Despite their strong sexual dimorphism, our aerodynamic model predicts that the flight performance of both sexes is remarkably similar. For example the maximum speed in sustained level flight is 28.1 m s^−1^ for the male and 29.2 m s^−1^ for the female (Fig. 3a), although the maximum terminal velocity of the female is 7.1 m s^−1^ higher than that of the male (111 vs 104 m s^−2^).

It is clear from Fig. 3a that only the rock dove and the mallard are expected to be able to outrun both sexes of peregrine in a sustained horizontal chase, and that in a vertical dive the falcon holds a significant speed advantage over all of the modelled prey (Fig. 3b). The ability of the falcon to outpace most of its prey in level flight is not sufficient to guarantee catch success in a horizontal chase, however, because our modelling shows that all of the prey species except the mallard can still escape by outmaneuvering the falcon. This can be seen in Fig. 3c–e, which shows that all of the modelled prey species except the Mallard have a higher maximum load factor, a higher maximum roll acceleration and a tighter minimum turn radius than the falcon when both are flying at their top sustainable horizontal speeds (ends of solid lines in (Fig. 3c–e). It follows that these prey species should always be able to outmaneuver a falcon, unless the falcon makes use of the absolutely higher maximum load factor and roll acceleration that it can achieve through the extra speed advantage gained by diving (dashed lines in (Fig. 3c–d). This is because, at their respective top sustainable horizontal flight speeds (ends of the solid lines in (Fig. 3c–d), the prey always have a higher maximum load factor and roll acceleration than the falcon, whereas the situation is reversed at their respective top dive speeds (ends of the dashed lines in (Fig. 3c–d). A downside of flying faster, however, is that increasing flight speed simultaneously increases a bird’s minimum turn radius (Fig. 3e). Thus although peregrines are able to match the load factor and roll acceleration of their prey by flying fast, they do so at the cost of having a larger minimum turn radius. A further complication is that any kind of maneuvering involves increased drag relative to non-maneuvering flight at the same speed, so that neither an erratically-flying prey species nor a falcon maneuvering in response to its prey will actually attain its top sustainable speed in a level chase. Moreover, prey species with very low aspect ratio wings, such as the blue tit, are expected to slow down more during maneuvers than species with very high aspect ratio wings such as the common swift, on account of their poorer lift-to-drag ratio. All things considered, catch success in our simulations is expected to be the result of a complex interplay between these various aspects of flight performance, together with the guidance behavior, response delays and visual error of the peregrine (Mills et al. 2018).

Our aerodynamic model also allows us to make quantitative predictions regarding the maximum load that both sexes of falcons can carry. We make these predictions by adding the mass and drag of the carried prey to that of the falcon, assuming that the falcon holds its prey facing forward with its wings retracted, such that the additional drag is just the parasite drag of the prey’s body. We then test, for each given prey species, whether there exists a flight speed at which the falcon is able to maintain level flight. In summary, both sexes of falcon are predicted to be able to carry all of the modelled prey species except the mallard in sustained level flight. Using allometric scaling to determine the body drag coefficient of the prey as a function of its mass (Mills et al. 2018), the male falcon is predicted to be able to carry 0.50 kg of prey, and the female 0.76 kg, which in each case is close to their own body weight. The female is therefore able to carry up to 50% heavier prey.

### Experiment 1: interspecific and intersexual variation in catch success

**Figure 4:**
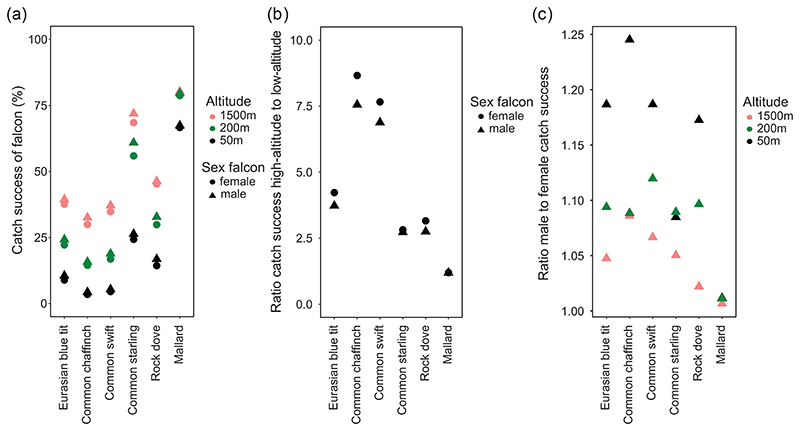
(a) Catch success of male and female peregrines hunting erratically maneuvering prey in experiment 1. Prey are ordered by size (frontally projected area), from small (left) to large (right). Falcons either dive from a high altitude above the prey (1500 m), reaching a high intercept speed (> 100 m s^−1^); from a moderate altitude (200 m), reaching a moderate speed (≈ 55 m s^−1^); or a low altitude (50 m), reaching approximately 35 m s^−1^. (b) The ratio of catch success of a falcon attacking from a high altitude to catch success of a falcon attacking from a low altitude. (c) Ratio of catch success of male falcons to females.

In the simulations for experiment 1, both sexes of falcon catch the mallard most frequently (Fig. 4a; see Supplementary material Appendix 1 Fig. A1 for the distributions of the minimum separation between falcon and prey during the encounters). This is to be expected given that the falcon

outperforms the mallard in every dimension of flight performance except maximum level airspeed (Fig. 3). After the mallard, catch success is next highest against the common starling, followed by the rock dove, blue tit and the common swift. Catch success is predicted to be lowest against the common chaffinch (Fig. 4a). Catch success for every prey species is highest in a high altitude attack, intermediate in an attack from moderate altitude, and lowest in an attack from low altitude. This makes sense in light of the falcon’s need to dive to obtain the speed advantage necessary to outmaneuver its prey. The gain in catch success that results from attacking from high altitude differs per prey species (Fig. 4b). For instance, in the case of the mallard, the ratio of catch success from a high altitude to catch success from a low altitude is only 1.2 for both sexes. Conversely, when either sex of falcon attacks a chaffinch, catch success is 7.5 times higher when attacking from high altitude than low. Again, this makes sense, because the falcon requires much more speed before it can outmaneuver its most maneuverable prey, whilst it need not fly fast to outmaneuver a mallard (Fig. 3c–e). Male falcons are found to be a small fraction better than females at catching most prey species, except the mallard, which they both catch equally well (Fig. 4c). Males have particularly higher catch success than females in attacks from low altitude, and the difference in catch success diminishes when both sexes attack from high altitude (Fig. 4c). In general, the smaller the prey, the greater the advantage of the male over the female, but the ratio is not monotonically increasing with decreasing prey size (follow triangles of one color from left to right in Fig. 4c). In summary, the results of experiment 1 demonstrate the importance of diving from a high altitude against all but the least maneuverable species of prey. They also show that the catch success of male falcons can be expected to be a little higher than that of females, on account of the higher maneuverability that the males display across their full range of flight speeds. Catch success is predicted to vary markedly between different prey species on account of differences in their flight performance. We explore which specific aspects of the flight performance of prey are key to these results in experiment 2.

### Experiment 2: why some prey species are harder to catch than others

**Figure 5:**
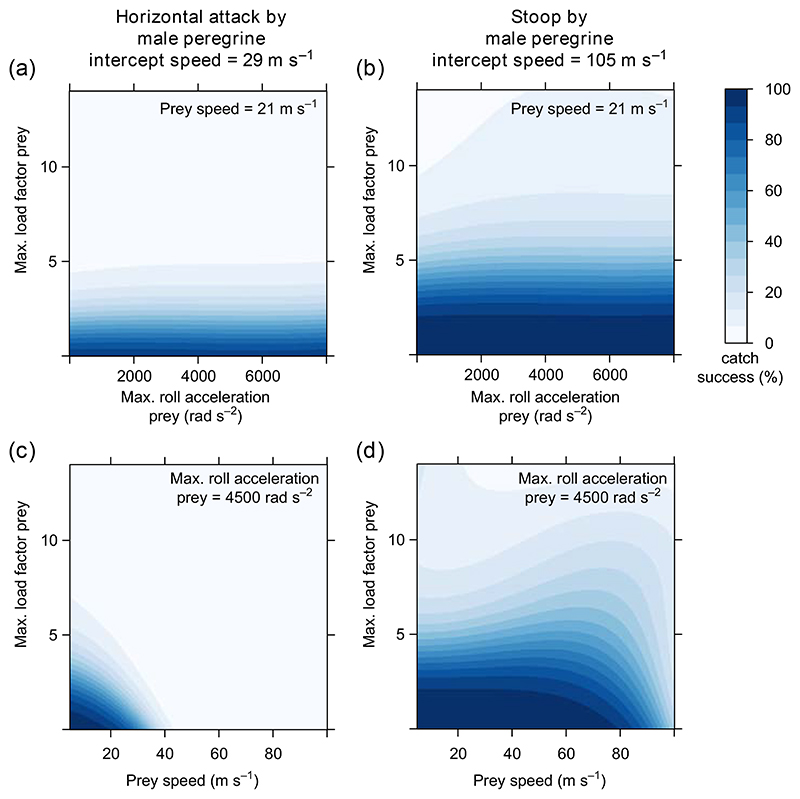
Attacks of male peregrines on artificial prey flying at a given constant speed and with artificially-set maximum load factor and roll acceleration (experiment 2). The catch success of the falcon is depicted by the blue gradient. The left column depicts horizontal attacks, and the right depicts dives by the falcon from high altitude (1500 m).

**Figure 6:**
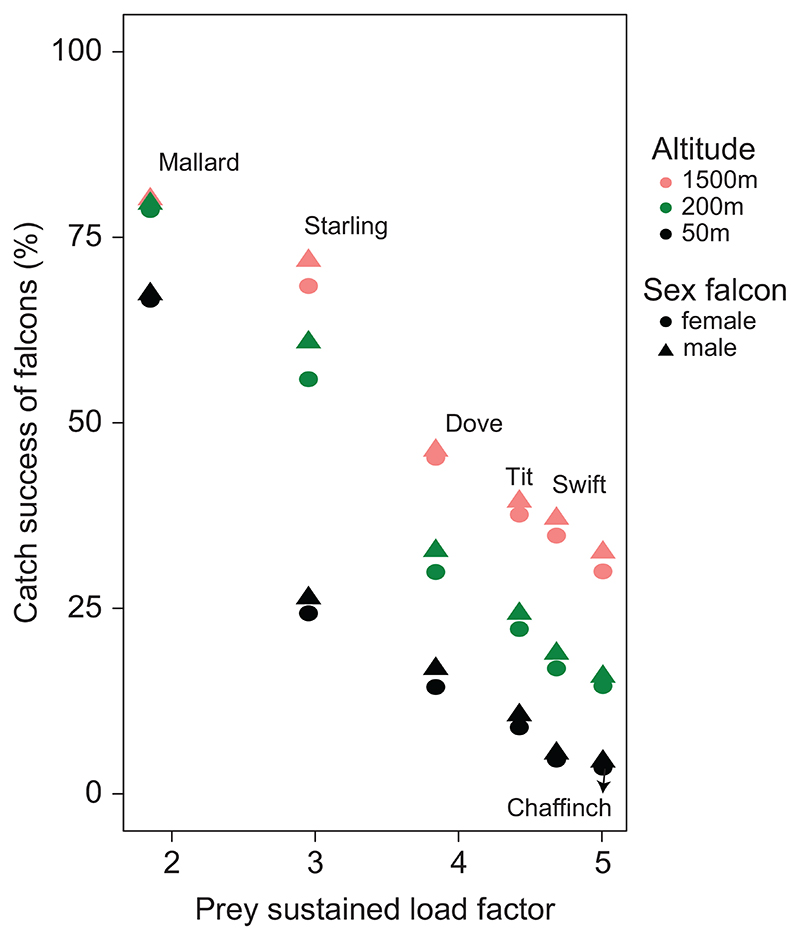
Catch success of model peregrines plotted against the mean load factor sustained by the different species of model prey during chases involving erratically maneuvering flight in experiment 1. Falcons either dive from a high altitude above the prey (1500 m), reaching a high intercept speed (> 100 m s^−1^), from a moderate altitude (200 m) reaching a moderate speed (≈ 55 m s^–1^) or a low altitude (50 m), reaching approximately 35 m s^–1^.

In experiment 2, we investigated which dimensions of the prey’s overall flight performance most impact the catch success of the falcon (see Methods: experimental setup). By artificially varying the maximum load factor (lift divided by weight) that the prey can exert, we find that the prey’s maximum load factor strongly affects the falcon’s catch success, both when the falcon attacks horizontally, and when it attacks in a high-altitude stoop (Fig. 5a–b). In particular, if the prey’s load factor matches or exceeds that of the falcon, then almost no prey are caught. The corresponding roll acceleration of the prey does not greatly affect the predator’s catch success (Fig. 5a–b). Prey speed influences catch success to a varying degree (Fig. 5c–d). When the falcon attacks horizontally, high prey speed decreases catch success – even when the prey’s speed remains lower than that of the falcon. When the falcon attacks from altitude in a high-speed stoop, only unrealistically fast prey are able to escape without exerting very high load factors. In the range of speeds at which real prey commonly fly (10–40 m s^−1^), those prey that exert very high load factors escape a stooping falcon more often when flying slowly (Fig. 5d), suggesting that a small minimum turning radius for the prey slightly diminishes the falcon’s catch success. Overall, we conclude that load factor (i.e. lift relative to body weight) is the primary dimension of flight performance that prey should maximize to evade capture by maneuvering. Other things being equal, a bird’s maximum attainable load factor increases with flight speed, but this is not the whole story, because exerting a high load factor increases drag, and the flight speed that a maneuvering bird reaches is therefore lower than its top speed in straight level flight. In testing whether load factor explains the differences in catch success found against the different prey species modelled realistically in experiment 1 (Fig. 3), it therefore makes sense to consider not the peak load factor attained by each prey species, but rather the mean load factor that it sustains over the course of a chase. Indeed, as Fig. 6 shows, the catch success of the falcon decreases monotonically with the prey’s mean sustained load factor across all of the different prey species that we examined, for every attack altitude and for both sexes of falcon. In summary, interspecific variation in the load factor that each prey species sustains during a chase is the primary driver of the interspecific variation in the falcon’s catch success.

### Experiment 3: why male falcons have higher catch success than females

**Figure 7:**
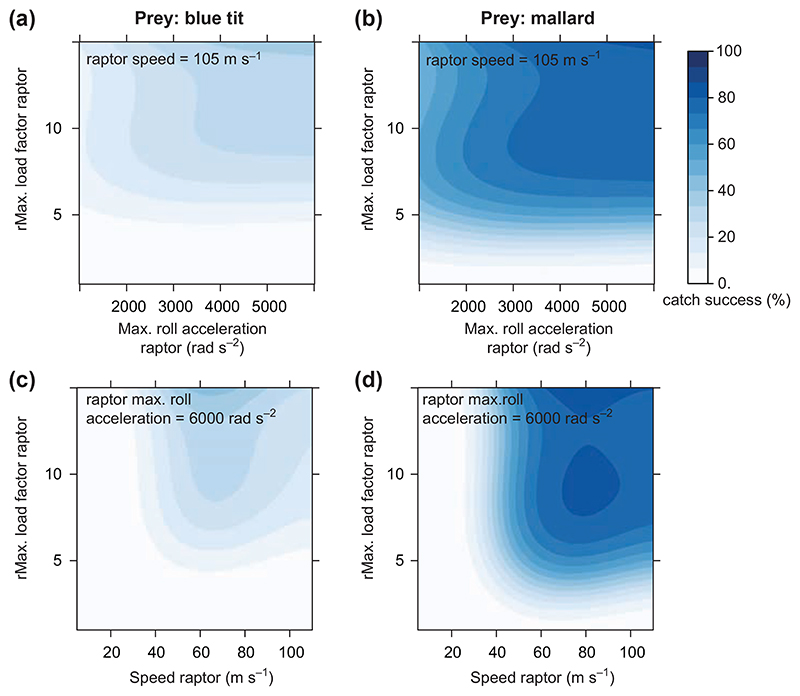
Attacks of generic raptors flying at a given constant speed and with artificially-set maximum load factor and roll acceleration (experiment 3). The left column depicts attacks on an erratically flying blue tit and the right column depicts attacks on an erratically flying mallard. The catch success of the raptor is depicted by the blue gradient.

Unsurprisingly, our generic raptor with fixed flight speed, fixed maximum load factor and fixed maximum roll acceleration has its highest catch success when it is able to exert both a high roll acceleration and a high load factor (Fig. 7a–b). This is true of simulated chases against both our smallest and our largest model prey species, the blue tit and the mallard.

All else being equal, our generic raptor also has a higher catch success when it flies at a moderately high speed (60–80 m s^−1^), rather than at a very high flight speed (> 80 m s^−1^; Fig. 7c–d). As we have shown elsewhere (Mills et al. 2018), very high flight speeds are important to falcons because of the higher load factors and higher roll accelerations that they enable, rather than because of the high speed of the motion per se. The physical relationship between these variables is deliberately broken in experiment 3, but the downsides of flying fast, such as the need to react quickly and accurately, are still captured by our model, which explains why increasing flight speed ultimately becomes detrimental. Taken together, these results show why the male falcon has a slightly higher overall catch success than the female. At any given airspeed, the maximum achievable load factor and maximum achievable roll acceleration are both higher for the male (Fig. 3c–d). At the point of intercept, a stooping female achieves almost the same load factor and roll acceleration as the male (Table 2), but only because she can dive faster (Fig. 3b–d). All else being equal, her higher flight speed is therefore expected to reduce catch success. These results also confirm why our model falcons maximize catch success in a high-speed stoop: by gaining speed, model falcons simultaneously gain a high load factor (Fig. 3c) and a high roll acceleration (Fig. 3d). Thus even though having a very high flight speed does not in itself increase catch success, it does enhance catch success in conjunction with the high load factor and roll acceleration that high airspeed enables.

## Discussion

### Factors affecting catch success

There have been remarkably few analyses to date of how flight performance affects catch success in aerial predators, but the case of Eleonora’s falcons *Falco eleonorae* attacking aerial prey was analysed by (Hedenström and Rosén 2001), who used Howland’s inequality (Howland 1974) to predict whether different potential prey species should be able to escape an attacking falcon. As we discussed in the Methods section, Howland’s inequality implies that prey can escape by out-turning a predator if and only if the magnitude of their centripetal acceleration exceeds that of their pursuer, which in turn implies that the load factor of the prey must exceed that of the predator. Applying this reasoning to our own model would lead to the conclusion that the prey in our simulations ought never to be able to out-turn a peregrine falcon that is able to reach a high flight speed by stooping, because the maximum achievable load factor of a peregrine is greater than that of any of the modelled prey. The fact that the prey often do escape in our simulations reflects the fact that there is rather more to attack and escape than is captured by Howland’s inequality, which merely sets the limits on escape by steady turning. Our detailed simulation approach incorporates a number of other important factors that determine success in attack and escape.

First, as shown by our previous work (Mills et al. 2018), response delays and errors in vision require the attacker to use an appropriately tuned guidance algorithm when intercepting prey. To achieve successful prey interception, the empirically and theoretically motivated guidance law that we use – pure proportional navigation – requires a low value of the navigation constant *N* that is optimized in relation to the mechanical and physiological properties of the falcon and the maneuverability of its prey (Fig. 2). The disadvantage of the low value of *N* that results from this optimization is that a very high load factor at least 2–3 times that of the prey is required to intercept a maneuvering target: a phenomenon that has also been well studied in missiles (Shneydor 1998). Second, in contrast to previous studies (Howland 1974, Hedenström and Rosén 2001), which assume that a tight turn is the optimal escape maneuver and ignore the unsteady flight dynamics, our modelling takes account of the fact that well-timed jinking maneuvers are the optimal evasive strategy against an attacker using proportional navigation (see Girard and Kabamba 2015 for jinking maneuvers against missiles, and see Mills et al. 2018 for a comparison of catch success of simulated falcons against smooth turning prey versus erratically jinking prey). Simply being able to achieve a large centripetal acceleration is not sufficient to allow an attacker to keep up with the unsteady maneuvers of a jinking target: the roll acceleration of the attacker is just as important in determining its catch success, because this determines how rapidly it can alter the direction of its centripetal acceleration. Third, in a prolonged chase, the mean sustained load factor becomes more important than the maximum attainable load factor. This is because of the increased drag that is produced when maneuvering, which reduces airspeed and thereby leads to a reduction in load factor. The complex dynamics of this behavior are evidently not captured by the steady-state approach adopted by Howland (1974) and others, but create an interesting trade-off with respect to the evolution of prey morphology: for instance, while a wing of low aspect-ratio may increase the instantaneous capacity of the prey to maneuver, by increasing wing area for a given span, a low aspect ratio wing also generates more drag, which causes the prey to slow down more when maneuvering, and thereby results in a decreased capacity of the prey to maneuver.

These and a number of other complexities are captured in detail by our simulation model, which incorporates many details of bird flight and matches empirical measurements of their flight performance closely (Mills et al. 2018). In summary, for an attacking falcon flying faster than its jinking prey, the key flight performance metrics affecting catch success are the falcon’s maximum load factor and maximum roll acceleration, and the mean load factor that the prey can sustain in jinking flight. As we have shown elsewhere (Mills et al. 2018), successful interception is only possible given precise vision, quick reactions and a suitably tuned guidance law. Nevertheless, even our detailed simulation model makes simplifying assumptions regarding the mechanics, aerodynamics, response delays and visual system of the birds. Our simulations therefore may not capture all of the important aspects of attack and escape, and revised theory in the future may shed light on new mechanisms that either complement or invalidate those described here. Additionally, the morphological parameters that are used to compute flight performance have varying degrees of measurement error associated with them. In the absence of any detailed knowledge of this measurement error, we are unable to place any quantitative uncertainty bounds on the estimates of flight performance in our model (but see Mills et al. 2018 for a sensitivity analysis to errors in reaction time, visual error or navigation constant). Nevertheless, any such inaccuracy in our quantitative modelling of flight performance should not affect qualitatively the general mechanisms described in this paper. Furthermore, any such inaccuracies are likely to hold equally for both male and female falcons, and their comparative flight performance should therefore be correctly described.

### Differences in catch success between sexes and across prey species

Previous authors have speculated that the sexual dimorphism of peregrines and other raptors has evolved so that a mated pair has a greater selection of prey to choose from Dekker (2009), Ratcliffe (2010). Male falcons are about two-thirds the size of females, and were therefore assumed to be more adept at catching small agile prey, while the greater muscle-mass of female falcons was supposed to allow them to carry less agile but heavier prey. Our modelling shows that female peregrines are indeed expected to be able to carry prey up to 50% heavier than that which the male can carry in level flight, but suggests that there are likely to be only relatively small differences between the sexes in their flight performance and their ability to catch agile prey, despite their large difference in size. While the female has a higher inertia to overcome when maneuvering, she also has a greater muscle mass, a larger wing area and a lower aspect ratio, and the net effect is that she is only slightly less agile than the male when flying at the same flight speed. The female is able to make up for this slight disadvantage by flying at a higher flight speed than the male, particularly when stooping, which allows her to increase the aerodynamic forces available for maneuvers. In our simulations, we find that the male falcon is slightly better at attacking agile prey in a low-level attack, but find that the difference in catch success diminishes when both sexes attack by stooping at high speed. Whether these small differences are ecologically important remains unknown, but because we find that both sexes are well capable of catching all of the prey species we studied, there is no exclusion of uncatchable species for the female. We therefore conclude that these differences in catch success are unlikely to be the sole explanation of why falcons have evolved to be sexually dimorphic.

On the other hand, their markedly higher ability to carry heavy prey in flight may well explain why females more often attack larger prey. Carrying prey in flight is important, because falcons are often harassed by kleptoparasitic raptors such as gyrfalcons *Falco rusticolus* or bald eagles *Haliaeetus leucocephalus*, especially when they consume their prey on the ground (Hector 1986, Dekker and Taylor 2005, Dekker 2009). Male peregrines are particularly vulnerable. While both sexes of peregrine may be able to fend off smaller kleptoparasites such as corvids, they risk losing their prey to others in the process. Hence, even when falcons are not breeding, they tend to bring most of their prey back to their perch, or even to consume their prey mid-air (Dekker 1988). The maximum load that a peregrine can carry has never been tested empirically, but only females have been observed to carry large prey such as mallards (Parker 1979, Dekker 1980, 1987). Our modelling suggests that a large mallard may be too heavy to carry even for a female peregrine, which would explain why larger ducks are often plucked and partially consumed by the female before being carried in flight (Dekker 2009). The advantage of the lighter male may be an energetic one. If catch success is comparable between the sexes, then it is reasonable to assume that the lighter male will consume less energy in attacking a specific prey species. In the absence of heavy prey, we should therefore expect the male to do most of the hunting. Empirical evidence for this suggestion is provided by Dekker and Taylor (2005), who studied attacks by pairs of falcons. Over 7 yr, the majority of observed solo hunts were performed by the male (432 hunts by the male versus 140 by the female), and while the male tended to specialise on catching small passerines, all of the gulls that it caught were immediately passed or dropped to the female or fledged juveniles, rather than being carried.

In general, we can expect that optimal prey choice will depend on the net gain in energy expected from a given species, which is a function of the predator’s expected catch success, the nutritional value of the prey, and the energetic cost of chasing and carrying the prey. Other things being equal, this suggests that falcons should prefer to hunt heavier prey, as our simulation results indicate that catch success for these species is generally higher, and their nutritional value is presumably higher. For example, the comparatively large rock dove has a lot of meat on it, and is relatively easy for both sexes to catch and carry, which in conjunction with its cosmopolitan abundance may explain why this is one of the most commonly taken prey species for both sexes of peregrine. As with most other species, catch success against this commonest of prey species is improved considerably by the higher flight speed and hence higher maneuverability achieved by stooping (Fig. 4a–b).

### Future work

We have focused here on studying the success of peregrines of both sexes hunting prey that maneuver erratically in mid-air, as this erratic flight behavior is widespread among alarmed prey. Our model assumes that the prey do not precisely time their erratic maneuvers, so it is likely that they could increase their success at evading the predator by timing escape maneuvers in response to its velocity and position. It will be interesting in future work to study escape behavior as a problem in optimal control. Specifically, given the attack strategy of the predator, and assuming full knowledge of its velocity and position, which escape strategies are optimal under the dynamics of bird flight?

## Supplementary Material

Supplementary Material Appendix 1

Supplementary Video

## Figures and Tables

**Table 1:** Morphological parameters of falcons and prey species. *f* = wingbeat frequency (Hz), *b* = wingspan (cm), *m_b_* = body mass (g), *m*_*w*_ = wing mass (g), *S* = wing area (dm^2^), AR = aspect ratio, *C*_*db*_ = body drag coefficient. References: (Pennycuick 1968, Hedenström and Møller 1992, Berg and Rayner 1995, Jenkins 1995, Tucker et al. 2000, Hedenstrom and Liechti 2001, Ward et al. 2001, 2004, Bruderer et al. 2010).

Common name	Species name	*f*	*b*	*m_b_*	*m_w_*	*S*	AR	*C_db_*
Peregrine falcon (male)	*Falco peregrinus*	5.1	87.3	528	32	8.97	8.49	0.16
Peregrine falcon (female)	*Falco peregrinus*	4.7	98.4	771	49	11.83	8.18	0.16
Eurasian blue tit	*Cyanistes caeruleus*	14.0	20.0	9.5	0.48	0.89	4.48	0.43
Common chaffinch	*Fringilla coelebs*	15.5	25.0	19.9	1.1	0.87	7.13	0.43
Common swift	*Apus apus*	8.3	39.2	42	2.1	1.57	9.81	0.35
Common starling	*5turnus vulgaris*	10.5	39.0	70	3.7	2.41	6.30	0.41
Rock dove	*Columba livia*	6.7	80.0	293	22.5	7.75	8.25	0.35
Mallard	*Anas platyrhynchos*	6.2	86.2	995	70	9.27	8.01	0.35

**Table 2:** Mean flight performance metrics in the simulations at the moment of intercept.

Species	Maneuver/attack	Speed (m s^−1^)	Load factor	Roll acc. (rad s^−2^)	Turn radius (m)
Eurasian blue tit	erratic	14.2	4.15	4695	3.7
Common chaffinch	erratic	23.8	4.75	4053	8.3
Common swift	erratic	24.7	4.51	3663	13.5
Common starling	erratic	20.2	3.23	2006	8.3
Rock dove	erratic	28.3	3.84	1961	20.2
Mallard	erratic	21.5	1.56	470	26.3
Male falcon	low-speed dive	38.1	4.63	2057	31.3
Male falcon	moderate-speed stoop	58.0	6.93	3034	48.6
Male falcon	high-speed stoop	102.1	11.06	5078	94.3
Female falcon	low-speed dive	38.3	4.41	1688	33.2
Female falcon	moderate-speed stoop	58.7	6.69	2523	51.5
Female falcon	high-speed stoop	107.0	11.15	4371	102.7
