# Supplementary material for: Sexual size dimorphism, prey morphology and catch success in relation to flight mechanics in the peregrine falcon: a simulation study
Source: J Avian Biol. Author manuscript; Available in PMC 2022 Jul 23. (PMC7613156; doi:10.1111/jav.01979)
Supplement: Supplementary Material Appendix 1 [file EMS145577-supplement-Supplementary_Material_Appendix_1.pdf]

Journal of Avian Biology

JAV-01979
Mills, R., Taylor, G. K. and Hemelrijk, C. K. 2019. Sexual size dimorphism, prey morphology, and catch success in relation to flight mechanics in the peregrine falcon: a simulation study. – J. Avian Biol. 2019: e01979

Supplementary material

Appendix 1

AI Video

Real-time graphical output of the flight simulator. The video shows example trajectories for each of the three attack conditions (attack from low, medium and high altitude) for a male falcon attacking a starling. It additionally shows the erratic flight path of the prey. The falcon's trajectory is depicted with a blue ribbon, and the prey's trajectory with a green ribbon. Further details are given in the top-right section of the video.

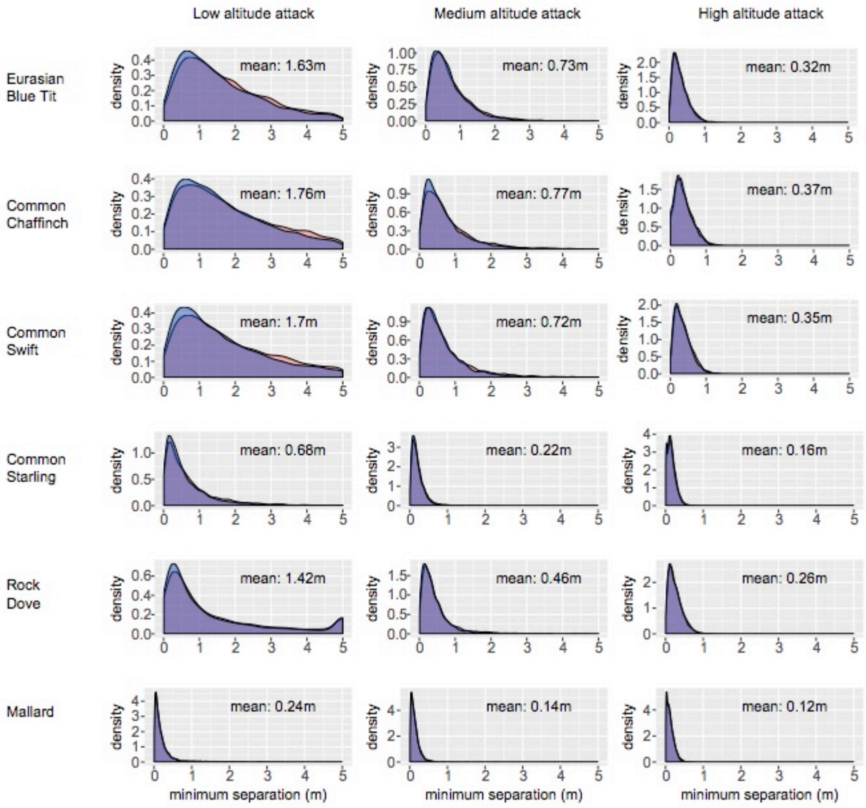

Figure A1

Means and distributions of the minimum separation distance between falcon and prey for each prey species and attack condition. The distribution of the male falcon is colored blue, the distribution of the female red, and the overlap between the distributions is purple. It can be seen that the distributions vary greatly between prey species and per attack condition, but are similar for male and female falcons. The female falcon is somewhat worse than the male in attacking highly maneuverable prey species (e.g. Blue Tit, Chaffinch, Swift) in a low altitude attack.

Supplementary Methods

Blade-element model

The assumptions, derivations, numerical schemes and solution methods and further details about the coefficients in the blade element model are provided in (Mills et al. 2018). Here, we succinctly describe the aerodynamic modeling applied to solve the objective function of maximizing speed, given a desired maneuver. We assume an elliptically shaped wing, for which the area of each section i across the wing length is

$$S_w^i = \frac{4\sqrt{(1 - i^2)}}{N\pi} S_w \tag{3}$$

where S_w is the wing area, and N the number of wing sections. For a sinusoidally flapping wing, the airspeed referenced to the wing section i at section j of the wing beat is

$$U^{ij} = \sqrt{(U_w^{ij})^2 + U_a^2} \tag{4}$$

$$U_w^{ij} = 2\pi f \sin\left(\frac{1}{2}\theta\right)\cos\left(\frac{j}{M}2\pi\right) l_w \frac{i}{N} \tag{5}$$

where U_w is the wing speed, U_a is the airspeed, f is the wingbeat frequency, θ is the angular stroke amplitude, and M the number of wing beat cycle sections. The following objective function is solved to estimate the forces on the birds. At each model time step, the bird maximizes its forward

acceleration by controlling the local angle-of-attack, and hence the coefficient of lift c_l at each section i of the wing and j along the wingbeat cycle:

$$\max_{c_l^{ij}} \frac{1}{2m} c_{d.b} S_b \rho U_a^2 + \sum_{j=0}^{M} \sum_{i=0}^{N} \left(\frac{U_w^{ij}}{U^{ij}} c_l^{ij} - \frac{U_a}{U^{ij}} \left(\frac{(c_l^{ij})^2}{\pi AR} + c_{d.f} \right) \right) \frac{S_w^i \rho (U^{ij})^2}{2Mm}$$

(6)

where $c_{d.b}$ is the body drag coefficient, m is the mass of the bird, S_b is the frontally projected wing area, ρ is the air density, AR is the aspect ratio of the wings, $c_{d.f}$ is the friction drag of the wings, S_w the wing area.

The above objective function is subject to the constraint that the exerted lift equates the magnitude of the force F^* dictated by its guidance law:

$$|F^*| = L = \sum_{j=0}^{M} \sum_{i=0}^{N} \left(\frac{U_a}{U^{ij}} c_l^{ij} + \frac{U_w^{ij}}{U^{ij}} \left(\frac{(c_l^{ij})^2}{\pi AR} + c_{d.f} \right) \right) \frac{S_w^i \rho (U^{ij})^2}{2M}$$

(7)

and the inequality constraint that the lift doesn't exceed the stall limit:

$$|c_l^{ij}| < c_{l.max}, \forall (i,j)$$

(8)

and that at no point throughout the wing beat the torque around the shoulder exceeds that which the muscles can hold

$$\left| \sum_{i=0}^{N} l_w i \left(\frac{U_a}{U^{ij}} c_l^{ij} + \frac{U_w^{ij}}{U^{ij}} \left(\frac{(c_l^{ij})^2}{\pi AR} + c_{d.f} \right) \right) \frac{S_w^i \rho (U^{ij})^2}{2M} \right| < \tau_{max}, \forall j$$

(9)

When the bird is flapping, f equates the maximum wing beat frequency of the bird, and while gliding $f = 0$ and the objective function is solved with respect to the local angle of attack and wing retraction ϕ, which alters the wing length ($l_w = \phi l_w^{max}$), wing surface area ($S_w = \phi S_w^{max}$), wing span ($b = 2l_w + l_b$, where l_b is the body width) and aspect ratio (b^2/S_w).

Rotation of model birds

For simplicity of the model, we assume that the bird's longitudinal axis is always aligned with its velocity vector. In the roll axis, the bird's rotational acceleration is limited by its whole body inertia and torque around its center of mass. Its whole body inertia is a function of the inertia of the body and of the inertia of the wings around the shoulder (see (Mills et al. 2018) for a derivation):

$$I = I_b + 2(I_{wing}\phi^2 + \frac{1}{4}0.098^2 m_b^{0.70} m_w + 0.098 m_b^{0.35} J\phi)$$

(10)

Where I is the total inertia around the center of mass along the roll axis, I_b the body inertia, I_{wing} the inertia of one wing around the shoulder, ϕ the proportion of wing retraction, m_w the mass of the wing, m_b the body mass, and $J = \sum_i m_i r_i$, where r denotes the distance from the bird's shoulder when wing is fully extended. The torque is a function of the differential lift force of the left and the right wing (equation 9 for each wing independently). By retracting its wings and changing the local angle-of-attack, the bird controls the roll acceleration. The bird rolls to manipulate the direction of its lift force, such that it aligns with the direction of the force dictated by the guidance law. A bang-bang control method is applied to determine the roll acceleration that aligns the lift force in the smallest amount of time.

The guidance law of falcons

The pure proportional navigation law that guides the falcon towards interception course is the following:

$$F^* = N m_f \frac{\overrightarrow{r_d} \times \overrightarrow{v_d}}{|\overrightarrow{r_d}|^2} \times \vec{v}_f - e_x \times m_f \vec{g} \times e_x$$

(11)

where $\vec{g}$ is the gravitational acceleration vector pointing downward, N is a free parameter called the navigation constant, $\overrightarrow{v_d}$ and $\overrightarrow{r_d}$ the difference in velocity and position between predator and prey, m_f the mass of the falcon, $\vec{e}_x$ is a unit vector along the longitudinal axis of the body of the falcon, and $\vec{v}_f$ the velocity of the falcon. F^* is the desired transverse force vector and the blade-element model determines the actual transverse force and corresponding forward acceleration that can be achieved. The setting of N that maximizes catch success depends on the speed, visual error and response delays of the predator, as well as the maneuvers of the prey (Mills et al. 2018). Therefore, this parameter is optimized in our simulations using genetic algorithms, maximizing catch success for each attack strategy and for each prey species (see **Methods: Genetic algorithms and Statistical Analysis**). We assume a small error by the falcon in the estimation of the angle of r_d (0.007 rad) and a response delay of $50ms$ between the current state of the simulation and the update of the aerodynamic forces (see (Mills et al. 2018) for the justification of this error and delay).

Prey maneuvers

Model-prey exert a sequence of hard-turns to random directions throughout their trajectory. Model-prey do not time their turns with respect to the oncoming falcon, because we assume that real prey are often unaware, or only partially aware, of the exact position and velocity of the falcon, and are

therefore not able to plan a reactive optimal evasive maneuver. The morphology of each prey species determines the magnitude of the forward acceleration, roll acceleration and transverse acceleration that they apply. Hence, we argue that the falcons' catch success while hunting various prey species in the model is a good indication of the real ability of both sexes of falcons to catch various maneuvering prey species.

The function for erratic prey motion is the following:

$$\vec{F}^{*} = m_p \vec{q}(i_{c_1}) c_2 a_{max} + m_p c_3 \kappa \vec{z} \tag{12}$$

where m_p is the mass of the prey, a_{max} the maximum achievable transverse acceleration, $\vec{q}(t)$ is a random unit vector, $\vec{z}$ a downward pointing unit vector and κ the current deviation from its initial altitude. $\vec{q}(t)$ is updated by the following equation:

$$\vec{q}(t+dt) = \begin{cases} \vec{q}(t), & \text{if } c_1 < i \sim U(0,1) \\ c_4 \vec{q}(t) + (1 - c_4)\vec{Q} & \text{otherwise} \end{cases} \tag{13}$$

where $\vec{Q}$ is a unit vector pointing towards a random azimuth angle and elevation between $-c_4$ and c_4 degrees. The parameters $c_1, \ldots, c_4$ determine the timing, direction and the magnitude of the exerted lift and roll acceleration. For each prey species, these four parameters are optimized, using genetic algorithms, to maximize the mean exerted transverse acceleration of the prey, subject to the constraint that the prey's altitude remains between -20m and 20m of its initial altitude.
